# Mesenchymal stem cells enhanced with SeNPs protection against CLP induction associated with liver injury are mediated *via* antioxidant, anti-inflammatory, and immunomodulatory activities

**DOI:** 10.3389/fimmu.2025.1602994

**Published:** 2025-09-05

**Authors:** Asmaa M. M. Abd El Aleem, Manal F. El-Khadragy, Ahmed E. Abdel Moneim, Sara H. Agwa, Fatma Abu Zahra, Mohga S. Abdalla

**Affiliations:** 1 Chemistry Department, Biochemistry Division, Faculty of Science, Helwan University, Cairo, Egypt; 2 Department of Biology, College of Science, Princess Nourah bint Abdulrahman University, Riyadh, Saudi Arabia; 3 Zoology and Entomology Department, Faculty of Science, Helwan University, Cairo, Egypt; 4 Al-Ayen Scientific Research Center, Al-Ayen Iraqi University, AUIQ, An Nasiriyah, ThiQar, Iraq; 5 Molecular Biology and Tissue Culture, Medical Faculty of Medicine Ain Shams Research Institute (MASRI), Ain Shams University, Cairo, Egypt

**Keywords:** sepsis-related liver injury, mesenchymal stem cells, selenium nanoparticles, inflammation, immunomodulation, NF-κB

## Abstract

**Introduction:**

Sepsis-induced liver injury is a serious issue in critical care. Since antibiotics are insufficiently effective to combat the disease and avoid upcoming organ failure, treatment with mesenchymal stem cells (MSCs) is an alternate strategy for treating liver damage. Thus, our work aimed to boost the therapeutic potential of MSCs by pretreating them with selenium in the form of sodium selenite (Na₂SeO₃) and selenium nanoparticles (SeNPs) in the cecal ligation and puncture (CLP) rat model of sepsis.

**Methods:**

Rats were split into groups that received MSCs alone, MSCs enhanced with Na₂SeO₃ (E1-MSCs), MSCs enhanced with SeNPs (E2-MSCs), antibiotics (Ab), and no therapy (CLP), in addition to the control and sham groups. Within 48 hours of the operation, liver tissues and blood samples were taken.

**Results:**

MSC treatment, significantly augmented with selenium compounds, markedly reduced markers of liver injury and signs of oxidative stress (MDA, MPO, NO) while elevating levels of GSH and antioxidant enzymes (GPx, GR, SOD, CAT). Furthermore, the therapies attenuated pro-inflammatory cytokines (TNF-α, IL-1β, IL-8) and inflammatory pathways (iNOS, MAPK9, NF-κB). Additionally, MSCs and enhanced MSCs improved hepatic tissue by alleviating the immunomodulatory indicators (COX-2, PGE2) and regulating apoptosis by raising (Bcl-2) and minimizing (Cas-3 and Bax). Histopathological analysis showed that MSC therapies, particularly when enhanced, restored the natural architecture of the liver.

**Discussion:**

This study concludes that MSCs enhanced with selenium compounds provide a promising therapeutic approach for liver dysfunction caused by sepsis, possibly through regulating antioxidants, anti-inflammatory processes, immunology, and hepatic tissue regeneration.

## 1 Introduction

Sepsis is a potentially lethal disorder that features organ failure resulting from an uncontrolled immunological defense to infection (1). It is a serious hazard to global health, accounting for 48.9 million new cases and 11 million deaths from sepsis each year, or 20% of all deaths worldwide (2). The sepsis pathophysiology includes alterations in the functions of several organs and uncontrolled infiltration of the body. This is caused by the *in vivo* release of significant amounts of inflammatory factors and neutrophil overactivation, leading to an inflammatory storm that ultimately results in edema formation, tissue damage, and organ dysfunction (3, 4). The liver, an essential organ that regulates immunity, metabolism, and detoxification, is especially susceptible to sepsis. Liver insufficiency, abnormalities in biochemical testing, and even liver failure can result from sepsis-related liver injury (SRLI) (5). Considering the severity of sepsis and its consequences on the liver, efficient treatments are urgently required.

Mesenchymal stem cells (MSCs) are currently an effective therapy for sepsis. According to Xiang et al. (6), these cells can differentiate and self-renew. They can also modulate inflammatory responses in liver tissue to produce an immunologically tolerant environment (7). Nonetheless, oxidative stress (OS) during cell culture and after transplantation restricts the curative effect of MSCs by reducing cell survival and functioning (8, 9). Increasing MSC survival and function with antioxidant supplementation has been the focus of recent research to solve this issue (10).

The antioxidative properties of selenium, a vital redox-active micronutrient, and its function in cellular defense processes attracted attention (11, 12). Selenium nanoparticles (SeNPs) are noteworthy because they have benefits over elemental Se, such as being more bioactive, soluble, and less poisonous (11). Additional benefits of nanosized selenium include its better antioxidant activity (13), involvement in enhanced glutathione peroxidase and thioredoxin reductase activity, and superior catalytic efficiency (14). Furthermore, Zhang et al. (15) research indicates that selenium deficiency is related to mortality risk in COVID-19 infection. Even though there may be advantages to combining MSCs and SeNPs, no study has explored how both work together to treat sepsis. This shortage in research offers an opportunity to explore a new therapeutic strategy that could enhance the effectiveness of MSC-based sepsis treatment. This work explores the possible therapeutic advantages of MSCs with SeNPs in a rat model of sepsis caused by cecal ligation and puncture (CLP). We hypothesize that MSCs combined with SeNPs will exhibit hepatoprotective and anti-inflammatory properties better than traditional MSC therapy.

## 2 Materials and methods

### 2.1 Ethical statement and experimental animals

Male adult albino rats (n = 41) from the experimental animal farm in El-Giza, Egypt, were used in the current study. The animals were kept in conventional circumstances in the Zoology and Entomology Department of Helwan University’s Faculty of Science. The study included 35 rats (n = 35, 200–220 g) divided randomly into 7 groups, five rats per cage, for endpoint experiments. We used an additional cage containing 6-week-old male rats for MSC isolation (n=6). The conditions included a 12-hour light/dark cycle, room temperature, unlimited access to water, and standard rat chow. A week before the trial, the rats were acclimated to the laboratory environment. Helwan University’s Institutional Animal Ethics Committee (permission No. HU-IACUC/Z/AEA0920-1) authorized all animal procedures, which adhered to the National Institutes of Health Guidelines for the Care and Use of Laboratory Animals, eighth edition.

### 2.2 Cecal ligation and puncture model

According to Fujimura et al. (16), sepsis was created utilizing the CLP model. In brief, rats received intraperitoneal thiopental sodium (25 mg/kg) anesthesia after a 12-hour fast. A 3-cm midline laparotomy was carried out, and a 4.0 silk thread was used to ligate the cecum beneath the ileocecal valve. An 18-gauge needle was used twice to puncture the ventral side of the cecum to enable the dispersal of fecal matter. After the cecum was repositioned into the intraperitoneal space, the abdomen was closed using 3.0 silk sutures.

### 2.3 Preparation and characterization of selenium nanoparticles

According to Khaled et al. (11), SeNPs were produced by an ordinary chemical reduction process, including sodium selenite (Na_2_SeO_3_) and ascorbic acid. A mixture of a 0.125 M ascorbic acid solution (10 mL) and an 8.6% bovine serum albumin (w/v, 5 mL) solution was combined, then a 20 mM sodium selenite solution (12.5 mL) was added dropwise. After homogenizing on a magnetic stirrer for 30 minutes at 1000 rpm, protecting from light, and storing in a refrigerator between 4 and 8°C, the color changed from translucent to brick red, confirming the ascorbic acid-mediated reduction of Se^4+^ to Se^0^. SeNPs were characterized by X-ray diffraction (XRD) (Bruker D2-Phaser) to examine the crystalline structure, phase distribution, and purity of the produced selenium nanoparticles; also, for particle size, size distribution, and zeta potential, dynamic light scattering (DLS) (Zetasizer Nano ZN, Malvern Panalytical Ltd., United Kingdom). DLS data are shown as the intensity-weighted Z-average diameter and polydispersity index (PDI), and the samples undergo triplicate analysis. Moreover, transmission electron micrographs were also captured using a high-resolution transmission electron microscope (TEM; JEOL Ltd., Japan) equipped with an electron diffraction pattern.

### 2.4 Bone marrow-derived mesenchymal stem cell isolation and culture

Tibiae and femurs were used to isolate BM-MSCs from male rats (n=6). Dulbecco’s Modified Eagle Medium (DMEM; GIBCO/BRL, Waltham, MA) supplemented with 13% fetal bovine serum (GIBCO/BRL) was used to flush the bone marrow. Using a density gradient method (Ficoll/Paque [Pharmacia]) to isolate nucleated cells, the cells were then grown as a primary culture in standard media (500 mL DMEM, 13% FBS, 1.5% penicillin/streptomycin, and 0.01% fungizone) for 12 or 14 days (GIBCO/BRL) at 37°C with 5% CO_2_. Every two to three days, the media were changed.

**Figure 1:**
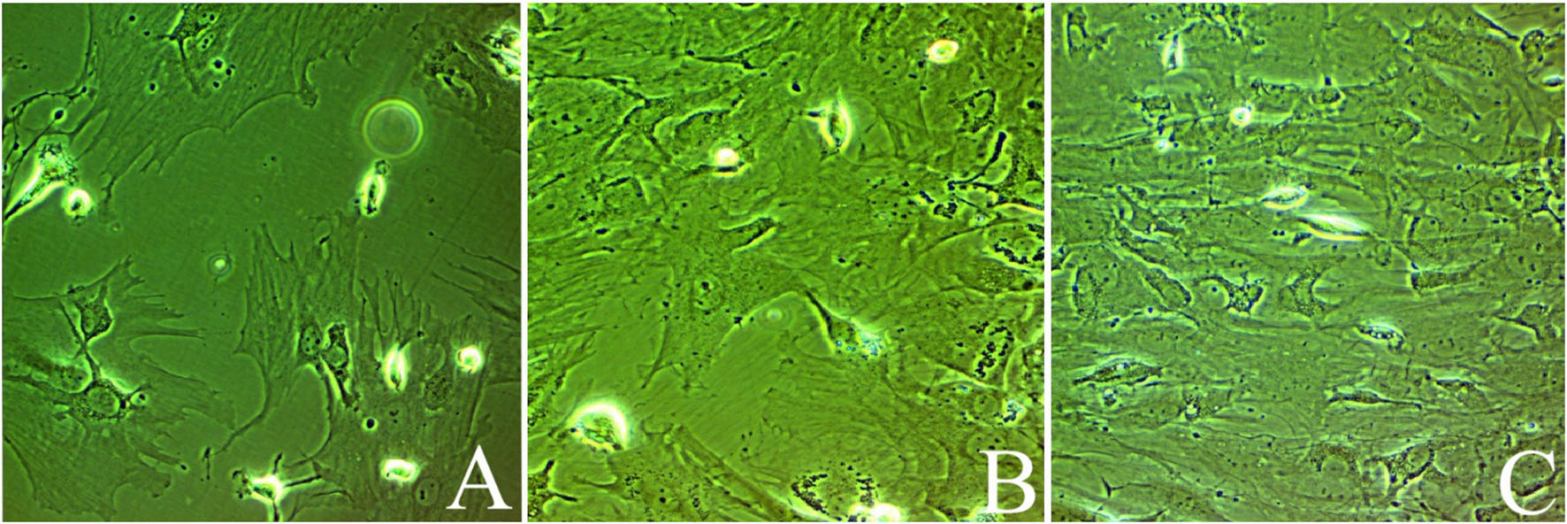
Bone marrow-derived mesenchymal stem cells at days of culturing: **(A)** Untreated MSCs, **(B)** mesenchymal stem cells enhanced with Na_2_SeO_3_ (E1-MSCs), and **(C)** mesenchymal stem cells enhanced with SeNPs (E_2_-MSCs). All photos were taken at 80% of confluency.

For enhancing MSCs, adherent cells at 60-70% confluency (second passage) were treated with either 100 nM sodium selenite (17) or 50 ng/mL SeNPs (10) until massive colonies developed, achieving a confluence of 80% to 90%. Cells were trypsinized by incubating with a mixture of trypsin (0.25%) and one mM ethylenediaminetetraacetic acid (EDTA; GIBCO/BRL) for five minutes at 37°C after two rounds of washing with phosphate-buffered saline. Cells were then immersed in media enriched with serum and kept in 50 cm² culture flasks for incubation (18). The adherent colonies were counted on day 14 (**Figure 1**). BM-MSCs were characterized by morphology and surface marker expression by flow cytometry using CD44-FITC (IM7, Cat#11-0441-82), CD45-PerCP-Cy5.5 (HI30, Cat #45-0459-42), and CD19-APC-Cy7 (SJ25-C1, Cat #A15429). Cells were analyzed using logarithmic SSC. At Mean Fluorescence Intensity (MFI) ≥4x baseline (Region ALL: X-Mean=1.03), positivity thresholds were established.

### 2.5 Animal groups and treatment protocol

The seven groups (n = 5 per group) of rats utilized in this investigation were randomly assigned. According to the study of Luo et al. (19), treatments were administered 3 hours post-CLP via intraperitoneal injection and then studied 48 hours later, as shown below:

*Control group:* Rats were given a single saline solution injection.

*Sham-operated group:* Rats were only given anesthesia to carry out the laparotomy and cecum manipulation operations; they were not exposed to CLP.

*Untreated CLP-induced septic (CLP) group:* Rats were subjected to CLP, left untreated for 48 hours to develop sepsis, and then euthanized without treatment.

*CLP-induced sepsis treated with bone marrow-derived mesenchymal stem cells (CLP-MSCs) group:* Rats were exposed to CLP to develop sepsis and then treated with 1 × 10^6^ BM-MSCs.

*CLP-induced sepsis treated with bone marrow-derived mesenchymal stem cells enhanced with Na_2_SeO_3_ (CLP+Na_2_SeO_3_+MSCs; CLP-E1-MSCs) group:* Rats were exposed to CLP to develop sepsis, then treated with 1 × 10^6^ Na_2_SeO_3_-enhanced BM-MSCs.

*CLP-induced sepsis treated with bone marrow-derived mesenchymal stem cells-enhanced with SeNPs (CLP+SeNPs+MSCs; CLP-E2-MSCs) group:* Rats were exposed to CLP to develop sepsis and then injected with 1 × 10^6^ SeNPs-enhanced BM-MSCs.

*CLP-induced sepsis treated with* a*ntibiotic (CLP-Ab) group:* Rats were exposed to CLP to develop sepsis and then treated with one dosage of ceftriaxone (100 mg/kg) (20).

### 2.6 Collecting and processing samples

Forty-eight hours after treatment, rats were euthanized by cervical dislocation and pentobarbital anesthesia (300 mg/kg, i.p.) in each group. For analysis, liver tissues and blood samples were collected. Three pieces of liver tissue were divided as follows:

The initial homogenate, 10% (w/v), was centrifuged for 10 minutes at 4°C at 3000 x g after being mixed with 50 mM Tris-HCl (pH 7.4) in an ice-cold solution. Biochemical analysis was then performed on the obtained supernatants. The second part was kept at -80°C for gene expression investigation. The remainder was stored in 10% neutral formalin for histological H&E and immunohistochemical studies.

### 2.7 Biochemical analysis

#### 2.7.1 Liver function parameters

The serum levels of alkaline phosphatase (ALP), aspartate aminotransferase (AST), and alanine aminotransferase (ALT) were measured using standard diagnostic kits.

#### 2.7.2 Markers of oxidative stress

The peroxidation of lipids was detected by measuring the level of malondialdehyde (MDA) in the liver (21). The O-dianisidine approach was used to measure MPO activity, a marker of leukocyte migration and aggregation (22). At 460 nm, spectroscopy was used to calculate the alteration in absorbance. Using the Ellman (23) approach, 5,5′-dithiobis (2-nitrobenzoic acid) was converted to a yellowish 5-thionitrobenzoic acid to quantify glutathione (GSH) spectrophotometrically at 405 nm.

#### 2.7.3 Estimation of antioxidant enzymes

Superoxide dismutase activity was assessed by determining how well it prevented phenazine methosulfate from reducing nitroblue tetrazolium dyeing (24). By detecting the rate of H_2_O_2_ breakdown at 240 nm, catalase activity was quantified (25). Ursini et al. (26) method was used to test glutathione peroxidase (GPx) activity indirectly through a coupled reaction with glutathione reductase (GR). In contrast, the activity of GR was calculated by measuring the reduction absorbance at 340 nm, which was caused by GR-catalyzed GSSG oxidation to NADP− in the presence of NADPH (27).

#### 2.7.4 Inflammatory biomarkers

Green et al. (28) used a colorimetric approach to estimate nitrite/nitrate (nitric oxide; NO) levels. TNF-α, IL-1β, and IL-8 levels in the liver tissue supernatant were measured using commercial ELISA kits as directed by the manufacturer. The sandwich ELISA technique measures NF-κB (Cat. no.: MBS287521).

#### 2.7.5 Assessment of immunomodulatory markers

The levels of PGE2 and COX-2 in the liver homogenates were evaluated according to the manufacturer’s instructions using commercially available ELISA kits (R&D Systems, Minneapolis, MN, USA).

#### 2.7.6 Measurement of apoptosis-related biomarkers

The ELISA kit’s instructions were followed, lysing both treated and untreated samples. The Bax and Bcl-2 proteins obtained from the cell lysate were coupled to the primary antibody, while HRP-conjugated secondary antibodies were used to detect them. The protein levels of Bax and Bcl-2 were then determined by measuring the absorbance at 450 nm using a microplate reader. To determine the signal transduction route causing apoptosis following the destruction of the liver, we investigated the activity of caspase-3 and how it inhibits apoptosis. The caspase-3 family, an essential player downstream of apoptotic death signaling, was measured using an ELISA kit per the guidelines set forth by the manufacturer.

### 2.8 Histological examination

Histological investigations were conducted to evaluate liver injury. Liver tissues were immersed in paraffin after being fixed in 10% buffered formaldehyde. These immersed tissues were sectioned (5 μm) and stained using eosin and hematoxylin to assess the general histological characteristics. A semi-quantitative approach was followed to score them. When evaluating liver tissues, we ranked the degradation of the hepatocytes and inflammation of the portals and lobules from 0 to 3. The tissue specimen’s score revealed the mean score across ten different fields. With a 100x magnification light microscope (Eclipse E200-LED; Nikon, Kawasaki, Japan). The severity of all pathological changes was graded semi-quantitatively, as previously recorded by Ahmed et al. (29). The pathological alterations investigated are inflammatory cell infiltration, disorganized hepatic strands, damaged hepatocytes, and pyknotic and apoptotic hepatocytes. H&E was evaluated on a scale of 0 to 3 by two blind investigators.

### 2.9 Immunohistochemistry examination

The immunological response of NF-κB and TNF-α was evaluated in hepatic tissue slices for all regimens. After deparaffinization, tissue blocks were hydrated again in ethyl alcohol and graded sequentially. The antigen was extracted using an EDTA (pH 8) solution, and endogenous peroxidases were inactivated with a five-minute incubation in a hydrogen peroxide solution. Next, the microscope slide was incubated with the primary monoclonal antibody against NF-κB p65 (2A12A7, Cat #33-9900) or TNF-α (28401, Cat #MA5-23720) for 60 minutes to avoid nonspecific binding, followed by washing with PBS. Next, the slide was incubated with anti-rat IgG, a second antibody, for 10 minutes (1:1000 dilution). Brown staining was visualized using the substrate solution containing diaminobenzidine (DAB), and it was counterstained for ten minutes using Mayer’s hematoxylin. The measurement of the positive immunoreactions was taken in ten non-overlapping high-power fields (40×) of paraffin sections of the liver for each group, utilizing ImageJ software (version 1.46, NIH, USA) and subsequently analyzed statistically.

### 2.10 Gene expression analyses

The expression level of inflammatory mediators (MAPK 9, iNOS, caspase-3, and Bax) was measured by quantitative real-time polymerase chain reaction (qRT-PCR). In brief, total RNA was isolated from the liver tissue using RNeasy Plus Mini kits. cDNA was synthesized using a Scripting cDNA synthesis kit (Bio-Rad, CA, USA) from 100 ng of total RNA, following the manufacturer’s instructions. qRT-PCR was performed using Power SYBR Green in Applied Biosystems 7500 (Applied Biosystems, USA), with duplicates for each reaction/sample. Gene primer sequences that were analyzed are included in **Table 1**.

**Table 1:** qPCR primer sequences.

Gene	Forward primer	Reverse primer
*GAPDH*	5′-GAAGGTCGGTGTGAACGGAT-3′	5′-ACCAGCTTCCCATTCTCAGC-3′
*MAPK9*	5′TCAGCACAGATGCAGCAGTAAG-3′	5′GTTTCATCGGCAGCCTTCCA3′
*iNOS*	5′-GTGAGGGGACTGGACTTTTAGAG-3′	5′-TCTCCGTGGGGCTTGTAGTT-3′
*Bax*	5′-AAGATGGGCTGAGGCTTCCT-3′	5′-CTTTCCCCGTTCCCCATTCA3′
*Caspase 3*	5′-GAGCTTGGAACGCGAAGAAA-3′	5′-TTGCGAGCTGACATTCCAGT-3′

### 2.11 Statistical analysis

The data met ANOVA assumptions (equal variances by the Brown-Forsythe test, p > 0.05; normality validated by the Shapiro-Wilk tests, p > 0.05). A one-way ANOVA was used with Tukey’s *post-hoc* test to analyze the data. The statistical significance level is p < 0.05; the results are presented as mean ± SD. GraphPad Prism 8 and SPSS v.20.0 were used for the analyses.

## 3 Results

### 3.1 Characterization of SeNPs

**Figure 2:**
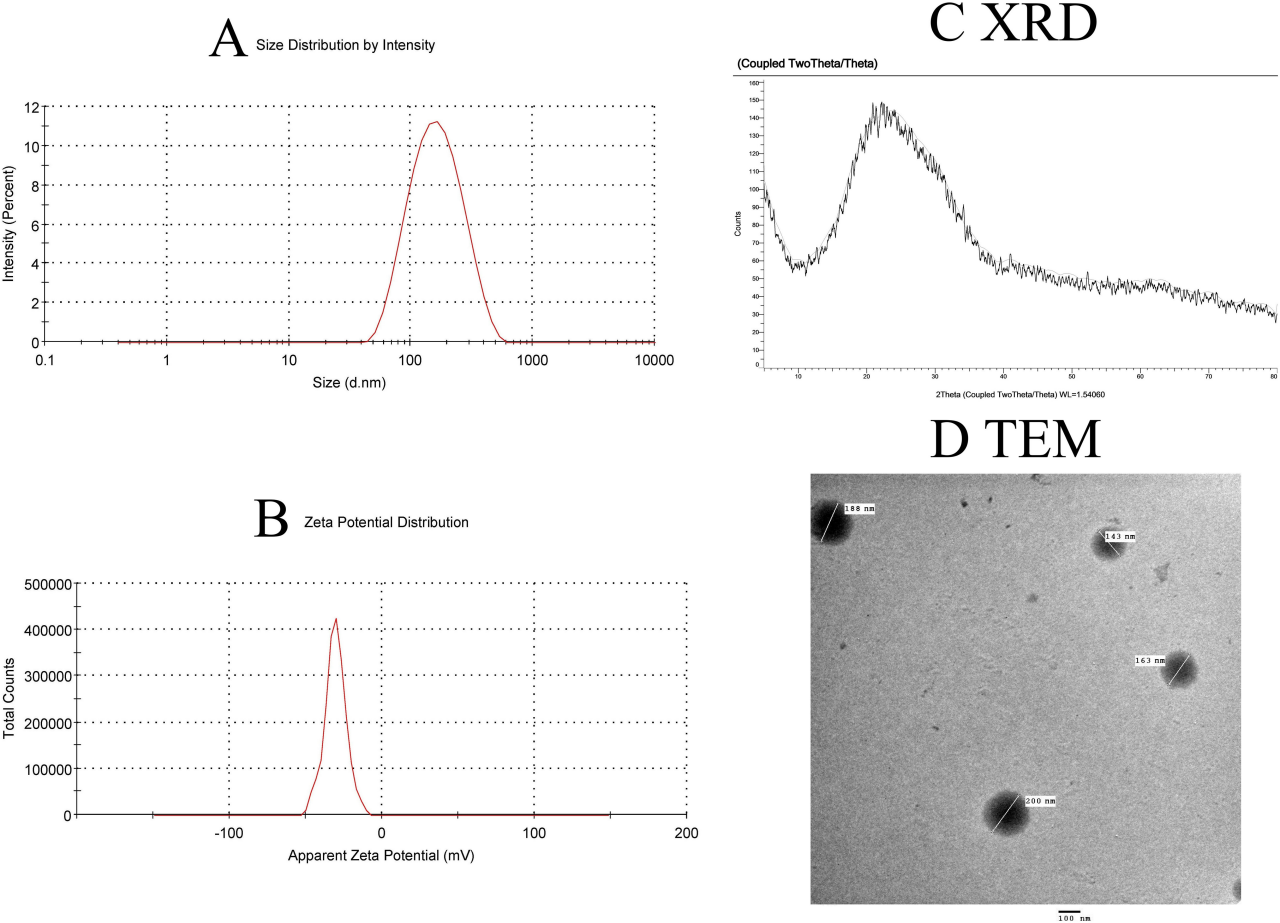
Characterization of SeNPs. **(A)** Intensity-weighted size distribution by dynamic light scattering (DLS), reporting Z-average diameter (262.5 nm) and polydispersity index (PDI = 0.068 ± 0.021, n=3). The logarithmic x-axis reflects the hydrodynamic size range. **(B)** Zeta potential distribution. **(C)** XRD pattern confirming crystalline structure. **(D)** TEM image confirming the revealed spherical particles with a diameter of less than 200 nm for the formed SeNPs.

A DLS analysis (**Figure 2A**) assessed the synthesized nanoparticles’ stability, size, and shape and showed a Z-average diameter of 262.5 ± 4.21 nm with a narrow polydispersity (PDI = 0.068 ± 0.021), confirming uniform SeNP synthesis. Zeta potential analysis indicated that the nano selenium particles had a zeta potential of 19.5 ± 0.11 mV, suggesting good stability (**Figure 2B**).

The crystalline structure of the produced Se nanoparticle was confirmed by XRD analysis. The crystalline structure of the produced Se nanoparticles is well-defined (**Figure 2C**). At 2̟ Ɵ values of 24.03, 30.3, 44.2, 52.2, 54.5, and 56.6, the sharp-edged peaks were visible. These results are consistent with Anu Mary Ealia and Saravanakumar (30), who used JCPDS No. 04–0783 as a reference number to show that the Se nanoparticles are crystalline, cubic, and free of these contaminants. The EM image revealed spherical particles with a diameter of less than 200 nm for the formed SeNPs. These particles were uniformly dispersed and showed no signs of agglomeration (**Figure 2D**).

### 3.2 Identification of BM-derived MSCs from rat

**Figure 3:**
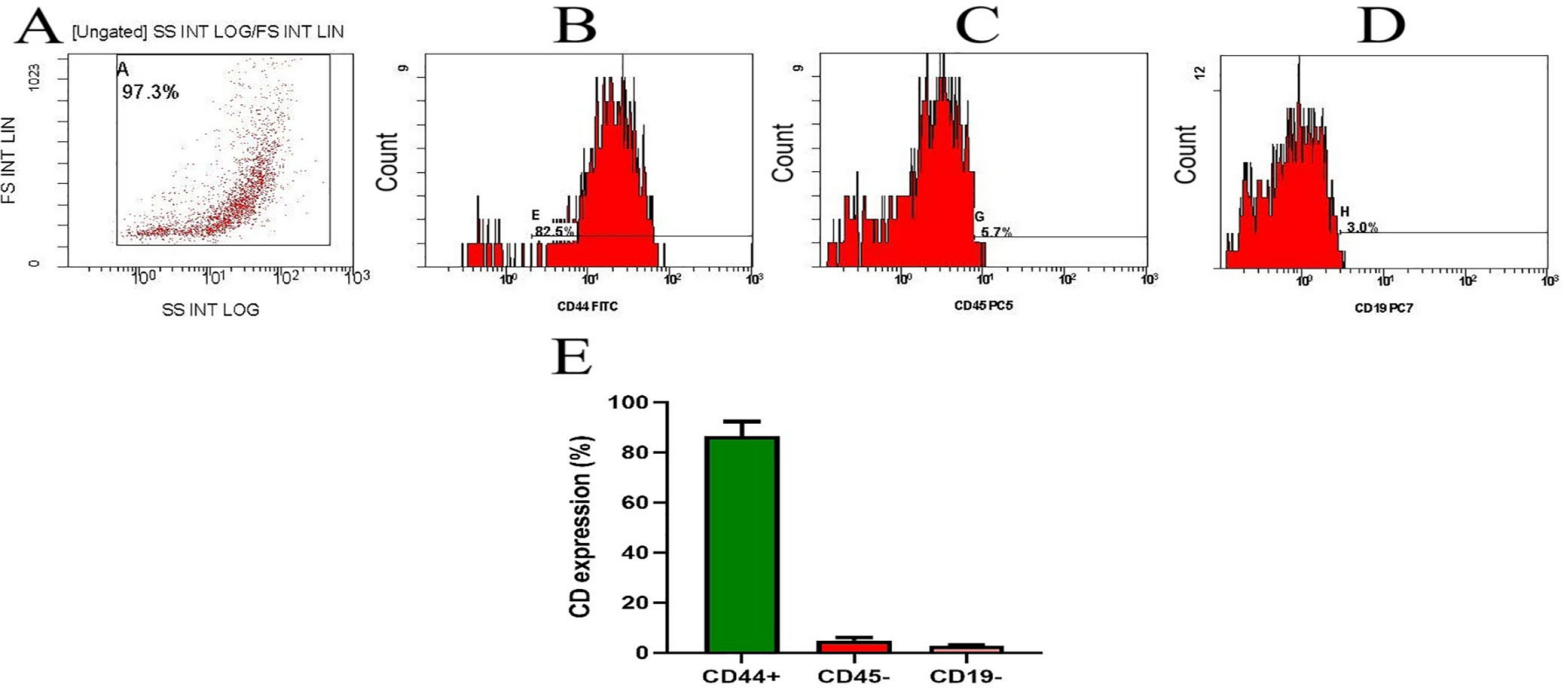
Flow cytometric characterization of bone marrow-derived MSCs. **(A)** Flow gate (logarithmic SSC vs. linear FSC). *Note.* Analysis of MSC cell surface marker expression. Cells were uniformly positive for **(B)** CD44 and negative for **(C)** CD45 and **(D)** CD19. **(E)** represents the CD percentage of two separate experiments and is presented as mean ± SD. Baseline MFI (Region ALL): 1.03.

During cell culture, BM-MSCs adhered to the plate. The cells’ morphology was spindle-shaped. The cells were screened before release and showed no signs of bacterial or fungal contamination. Before cell injection, the mean cell viability was 92.2% ± 2.5. Flow cytometry was used to analyze the immunophenotype of BM-MSCs. As illustrated in **Figure 3**, BM-MSCs exhibited substantial positive results for mesenchymal stem cell-specific markers, such as CD44 (82.46%, MFI=28.7), but negative results for CD45 (5.73%, MFI=10.9) and CD19 (2.96%, MFI=4.04). Specificity was confirmed by CD44+ cells, which displayed an MFI that was 28 times greater than baseline (28.7 vs. 1.03).

### 3.3 The potential effect of enhanced MSCs on CLP-induced liver injury in rats

**Figure 4:**
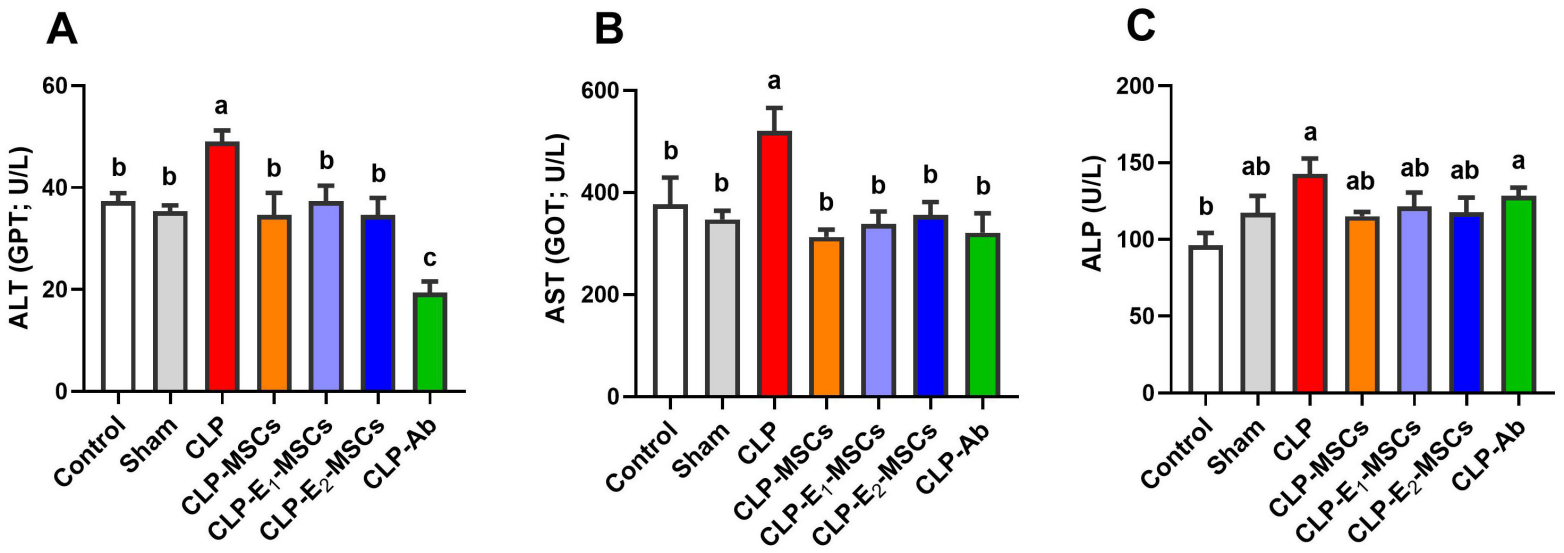
Effects of mesenchymal stem cells (MSCs), mesenchymal stem cells enhanced with Na_2_SeO_3_ (E_1_-MSCs), mesenchymal stem cells enhanced with SeNPs (E_2_-MSCs), or standard treatment antibiotics **(Ab)** on liver function tests in the serum of septic rats. **(A)** alanine aminotransferase (ALT), **(B)** aspartate aminotransferase (AST), and **(C)** alkaline phosphatase (ALP). The mean ± SD data displays the biochemical results (n = 5). Different letters show statistically significant differences between groups by one-way ANOVA, with Tukey’s test at P < 0.05. Groups with the same letter have no significant differences.

Serum levels of ALT, AST, and ALP were used to assess the extent of liver damage brought on by CLP. A significant elevation in blood levels of ALT (P < 0.0001, F (6, 28) = 49.74), AST (P < 0.0001, F (6, 28) = 21.69), and ALP (P < 0.0001, F (6, 28) = 14.01) was observed 48 hours after CLP surgery compared to the control group. There is a significant reduction in the blood levels of liver function enzymes in the treatment groups (MSCs, E_1_-MSCs, E_2_-MSCs, and Ab) as compared to the CLP group (P < 0.05, **Figure 4**). While there is no significant difference between the blood levels of alkaline phosphatase in the CLP group and the rats treated with antibiotics, the other treated groups exhibit modest significance.

### 3.4 Synergistic effects of MSCs only or with enhancement on redox status of damaged liver in septic rats

**Figure 5:**
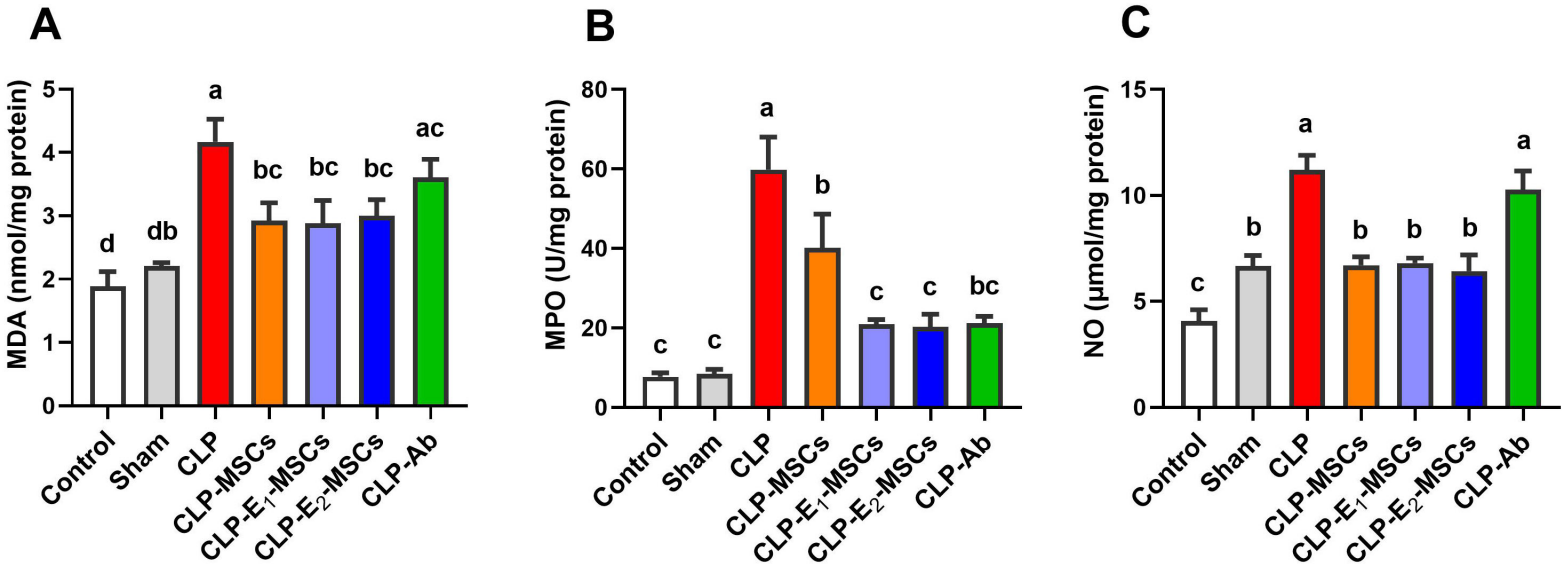
Effects of mesenchymal stem cells (MSCs), mesenchymal stem cells enhanced with Na_2_SeO_3_ (E_1_-MSCs), mesenchymal stem cells enhanced with SeNPs (E_2_-MSCs), or standard treatment antibiotics (Ab) on oxidative stress markers in damaged liver tissue of septic rats. **(A)** malondialdehyde (MDA), **(B)** myeloperoxidase (MPO), and **(C)** nitric oxide (NO). The mean ± SD data displays the biochemical results (n = 5). Different letters show statistically significant differences between groups by one-way ANOVA, with Tukey’s test at P < 0.05. Groups with the same letter have no significant differences.

**Figure 6:**
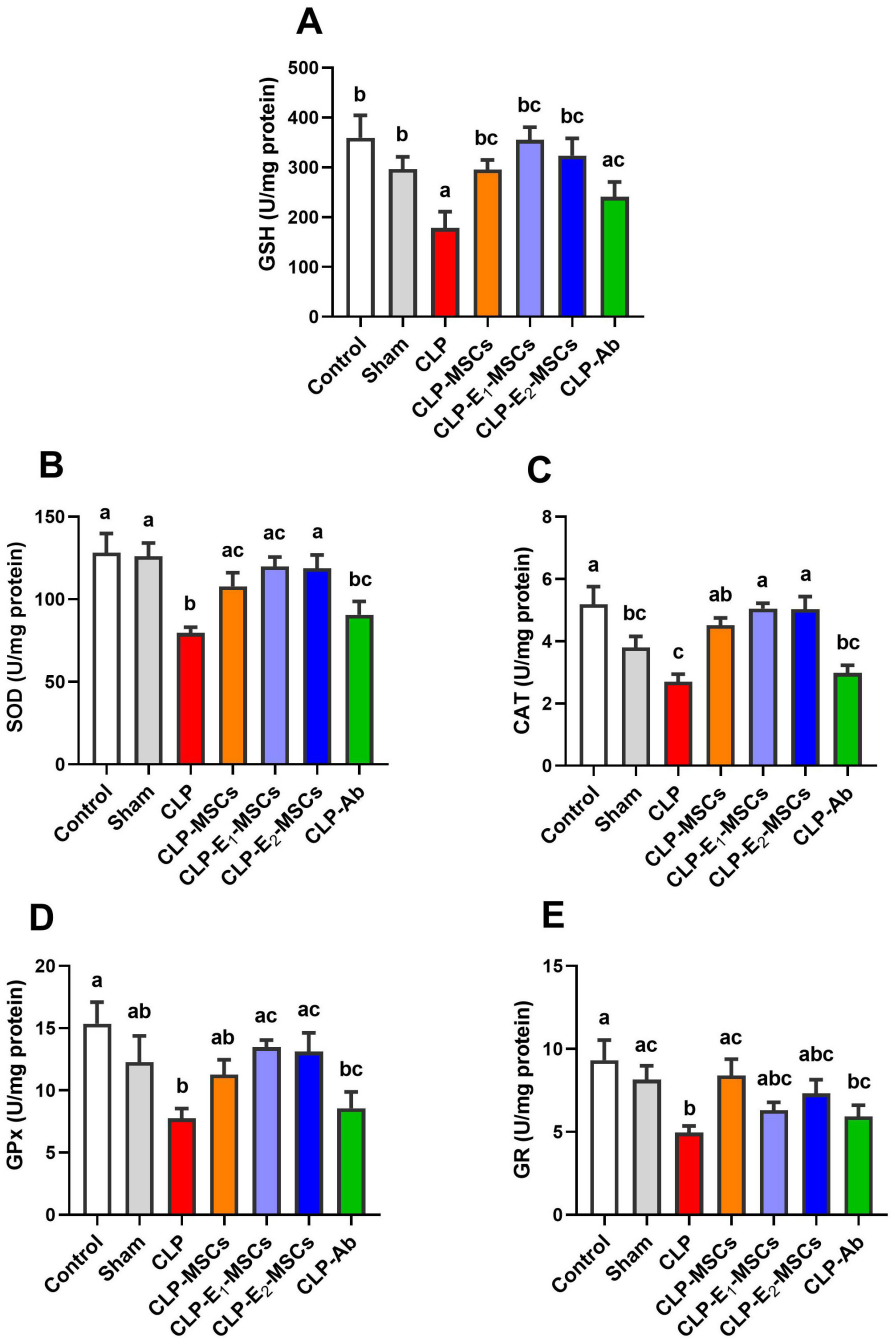
Effects of mesenchymal stem cells (MSCs), mesenchymal stem cells enhanced with Na_2_SeO_3_ (E_1_-MSCs), mesenchymal stem cells enhanced with SeNPs (E_2_-MSCs), or standard treatment antibiotics (Ab) on the antioxidant enzymes in damaged liver tissue of septic rats. **(A)** reduced glutathione (GSH), **(B)** superoxide dismutase (SOD), **(C)** catalase (CAT), **(D)** glutathione peroxidase (GPx), and **(E)** glutathione reductase (GR). The mean ± SD data displays the biochemical results (n = 5). Different letters show statistically significant differences between groups by one-way ANOVA, with Tukey’s test at P < 0.05. Groups with the same letter have no significant differences.

The effects of sepsis induction and the ameliorating effects of MSCs, with or without augmentation, on the redox status within the liver’s tissue are illustrated in **Figures 5** and **6**. According to the results, the model group’s liver tissue had significantly higher levels of hepatic lipoperoxidation in terms of MDA [P < 0.0001, F (6, 28) = 39.43], MPO [P < 0.0001, F (6, 28) = 76.48], and NO [P < 0.0001, F (6, 28) = 80.96] than the control group. Alongside this, the CLP group’s GSH [P < 0.0001, F (6, 28) = 21.68] content and the activity of antioxidant enzymes [SOD (P < 0.0001, F (6, 28) = 27.26), CAT (P < 0.0001, F (6, 28) = 45.47), GPx (P < 0.0001, F (6, 28) = 18.83), and GR (P < 0.0001, F (6, 28) = 17.57)] significantly decreased in comparison to the control group. In contrast to the untreated CLP group, MSCs, E_1_-MSCs, E_2_-MSCs, or Ab treatments markedly (P < 0.05) increased these hepatic tissue-resident antioxidant enzymes. However, antibiotic treatment exhibits the least significance compared to other treated groups. MSCs inhibited oxidative damage significantly (P < 0.05) by lowering liver MDA, MPO, and NO levels alone or in combination with enhancers. In contrast, compared to the control rats, antibiotic therapy was unable to reduce these CLP-induced alterations in oxidative damage, particularly with regard to the NO level. This outcome suggests that the advantages of antibiotics are limited, even though MSCs protect the liver from oxidative stress, and MSC therapy might better treat oxidative damage in hepatic tissue after CLP-induced injury.

### 3.5 The combined therapy of MSCs and SeNPs boosted the damaged hepatic tissue’s anti-inflammatory and immunomodulatory response

**Figure 7:**
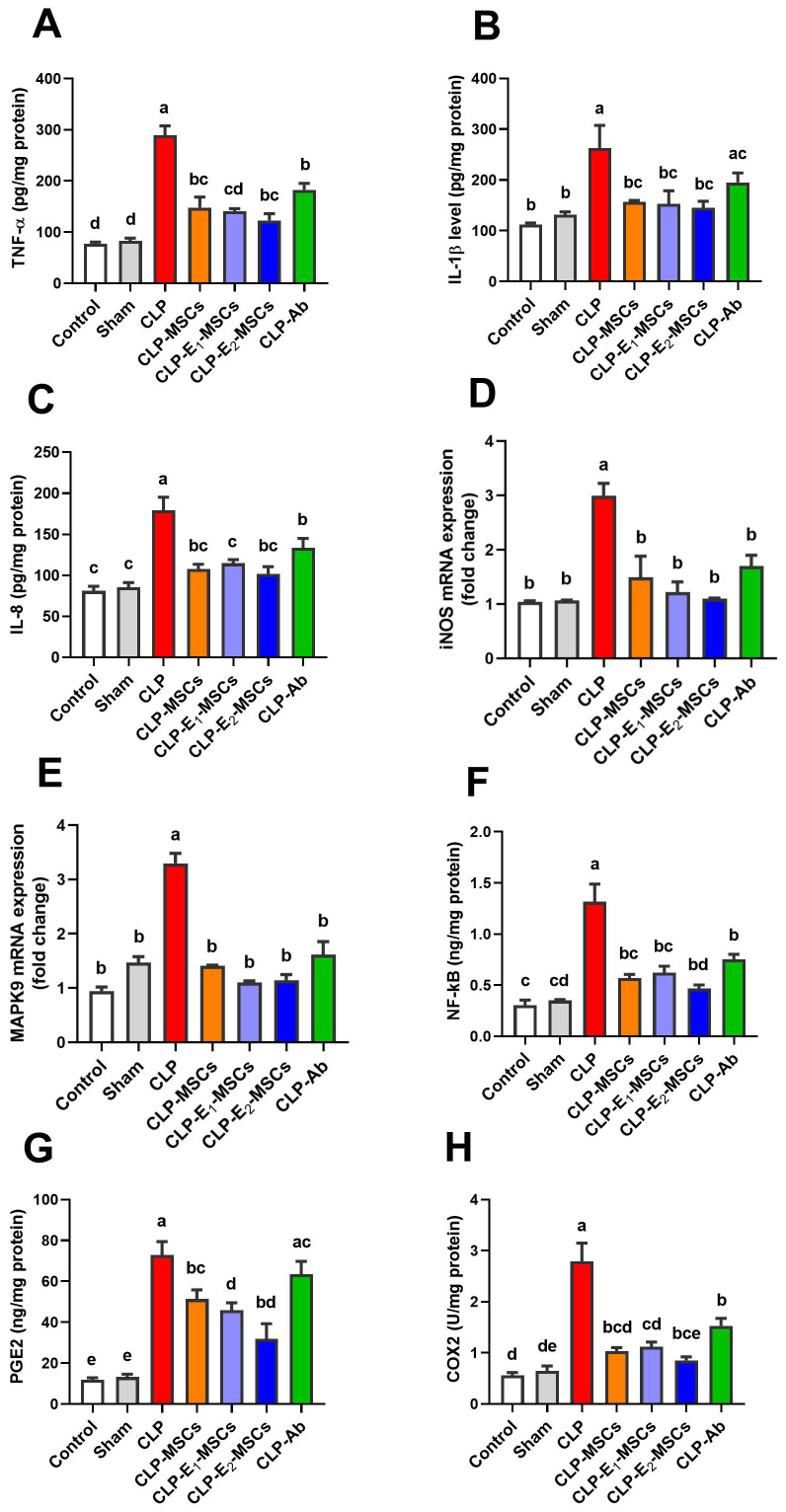
Effects of mesenchymal stem cells (MSCs), mesenchymal stem cells enhanced with Na_2_SeO_3_ (E_1_-MSCs), mesenchymal stem cells enhanced with SeNPs (E_2_-MSCs), or standard treatment antibiotics (Ab) on the inflammatory and immunomodulatory biomarkers in damaged liver tissue of septic rats. **(A)** tumor necrosis factor-alpha (TNF-α), **(B)** interleukin-1β (IL-1β), **(C)** interleukin-8 (IL-8), **(D)** inducible nitric oxide synthase (iNOS), **(E)** mitogen-activated protein kinases 9 (MAPK9), **(F)** nuclear factor kappa B (NF-κB), **(G)** prostaglandin E_2_ (PGE2), and **(H)** cyclooxygenase-2 (COX-2). The mean ± SD data displays the biochemical results (n = 5). Different letters show statistically significant differences between groups by one-way ANOVA, with Tukey’s test at P < 0.05. Groups with the same letter have no significant differences.

This research examined the impact of MSCs alone or enhanced with Na_2_SeO_3_ or SeNPs on inflammatory and immunomodulatory mediators in CLP rats (**Figure 7**). A significant increase in the levels of pro-inflammatory cytokines [TNF-α (P < 0.0001, F (6, 28) = 157.50), IL-1β (P < 0.0005, F (6, 28) = 8.45), and IL-8 (P < 0.0001, F (6, 28) = 69.56)], along with cytokine-induced upregulation of iNOS (P < 0.0001, F (6, 14) = 36.48), was observed in hepatic tissues of the experimental CLP group compared to the control group. However, treatment with MSCs, either alone or combined with Na_2_SeO_3_ or SeNPs, significantly subsided (P < 0.05) these mediators and tissue inflammatory reactions in the CLP model group. The anti-inflammatory mechanism of MSCs on CLP-induced liver damage was confirmed by inhibiting the MAPK9 signaling pathway. In addition, NF-κB is another key marker for clarifying the efficacy of MSCs in regulating inflammation in sepsis. The study revealed that MAPK9’s mRNA expression was significantly upregulated (P < 0.0001, F (6, 14) = 110.0), with a further notable rise in the NF-κB (P < 0.0001, F (6, 28) = 97.50) levels in inflamed hepatic tissue of the CLP group compared to control. On the contrary, the CLP groups that received different treatments, either MSCs, E_1_-MSCs, or E_2_-MSCs, displayed marked downregulation of MAPK9 expression and decreased (P < 0.05) values of NF-κB. Meanwhile, the liver tissue’s immunomodulatory circumstances in reaction to MSCs alone and SeNPs in septic model rats were verified by measuring the levels of PGE2 and COX-2. Results revealed that the CLP group has higher levels of PGE2 (P < 0.0001, F (6, 28) = 104.21) and COX-2 (P < 0.0001, F (6, 28) = 114.20) than the control group, but in comparison to the untreated CLP group, treated CLP animals with various therapies showed a significant (P < 0.05) decrease in these markers. Therefore, MSCs, particularly when incorporated with SeNPs, may be vital in suppressing liver inflammation and improving overall outcomes. Therefore, combined therapy of MSCs and SeNPs could be an intriguing strategy for curing inflammatory and immunomodulatory hepatic diseases.

**Figure 8:**
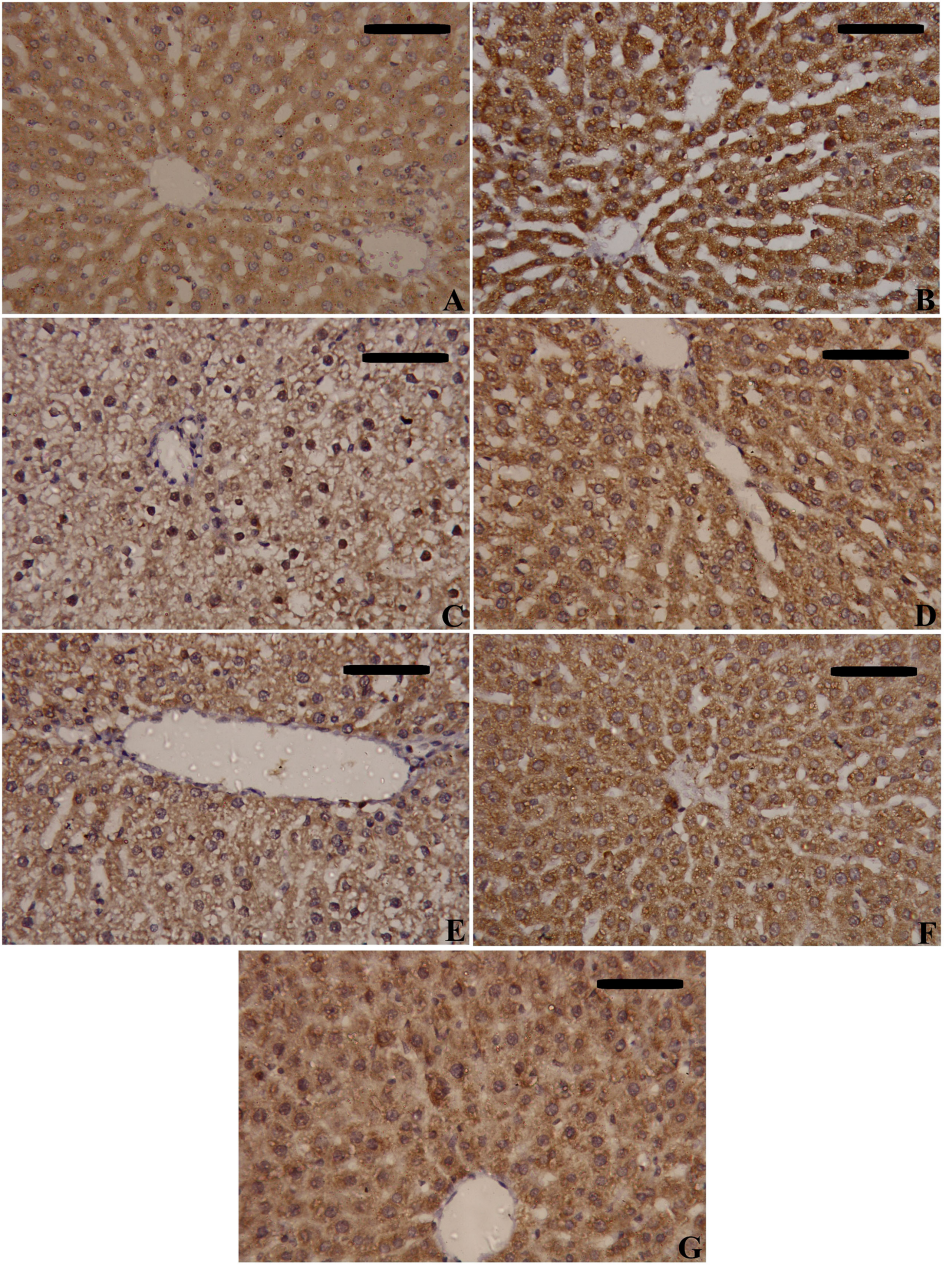
Effects of mesenchymal stem cells (MSCs), mesenchymal stem cells enhanced with Na_2_SeO_3_ (E_1_-MSCs), mesenchymal stem cells enhanced with SeNPs (E_2_-MSCs), or standard treatment antibiotics (Ab) on the inflammatory biomarkers’ immunohistochemistry analysis of nuclear factor kappa B (NF-κB). **(A)** Control; **(B)** Sham; **(C)** CLP; **(D)** CLP-MSCs; **(E)** CLP-E1-MSCs; **(F)** CLP-E2-MSCs; **(G)** CLP-Ab. Scale bar = 100 µm.

**Figure 9:**
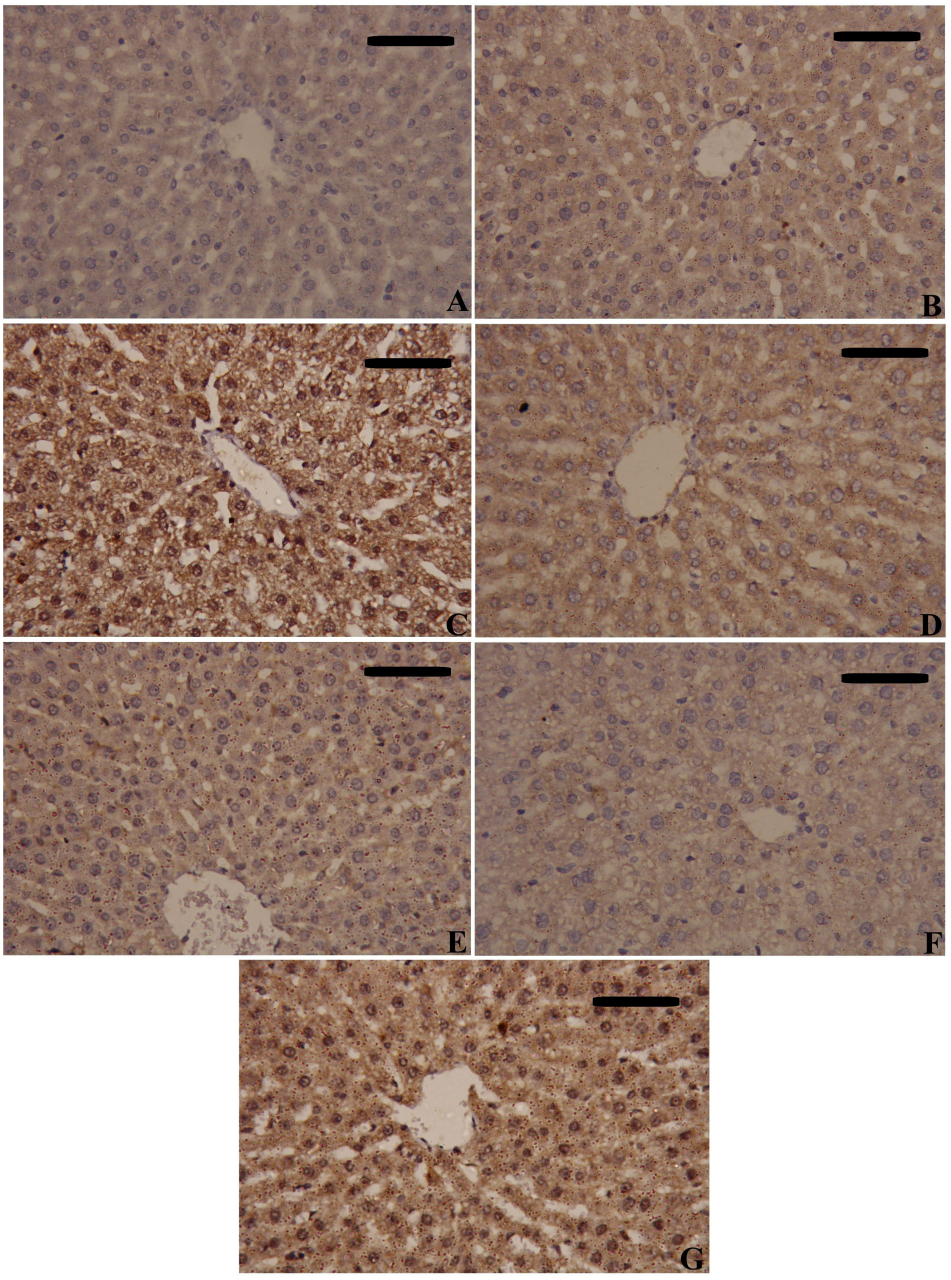
Effects of mesenchymal stem cells (MSCs), mesenchymal stem cells enhanced with Na_2_SeO_3_ (E_1_-MSCs), mesenchymal stem cells enhanced with SeNPs (E_2_-MSCs), or standard treatment antibiotics (Ab) on the inflammatory biomarkers’ immunohistochemistry analysis of tumor necrosis factor-alpha (TNF-α). **(A)** Control; **(B)** Sham; **(C)** CLP; **(D)** CLP-MSCs; **(E)** CLP-E1-MSCs; **(F)** CLP-E2-MSCs; **(G)** CLP-Ab. Scale bar = 100 µm.

**Figure 10:**
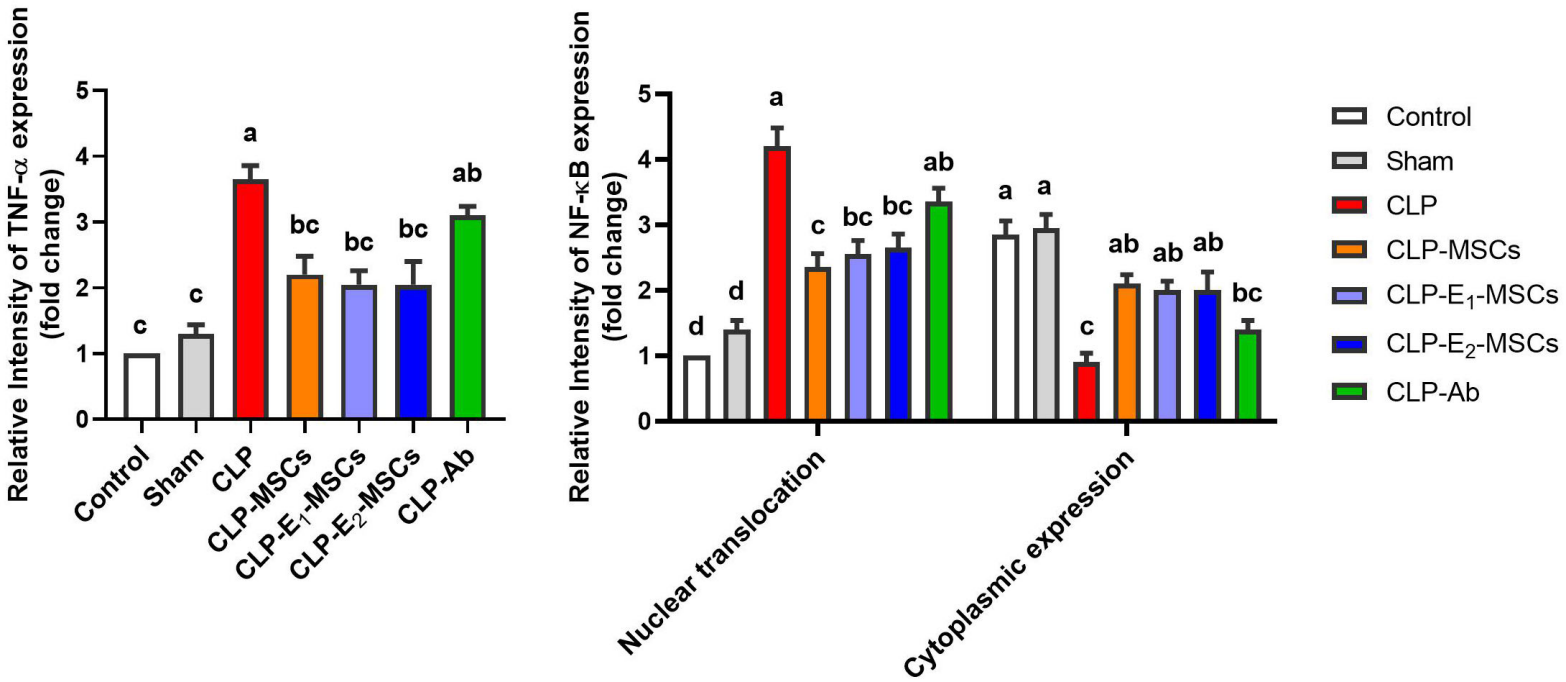
Quantitative analysis of tumor necrosis factor-alpha (TNF-α) and nuclear factor kappa B (NF-κB) either cytoplasmic or nuclear localization immunoreactions in the hepatic tissues of the different studied groups. The mean ± SD data displays the biochemical results. Different letters show statistically significant differences between groups by one-way ANOVA, with Tukey’s test at P < 0.05. Groups with the same letter have no significant differences.

NF-κB and TNF-α immunostaining were performed to predict whether treatments with MSCs alone or with enhancement can spare hepatic cells from inflammation alongside necrosis induced by CLP. As shown in **Figures 8**–**10**, our results revealed that the inflamed hepatic cells had intense dark brown positivity staining for both NF-κB (**Figure 8**) and TNF-α (**Figure 9**) in the septic group with respect to the control group, indicating significant inflammation. Nuclear localization (**Figure 10**) was still discernible, despite the nucleus being less visible due to the dark stain. However, the control group lacked nuclear NF-κB and only showed light brown cytoplasmic staining. On the contrary, hepatic cells of the treated groups, either with MSCs alone or with enhancement, significantly decreased staining intensity, with NF-κB predominantly localized in the cytoplasm and distinctly visible blue nuclei, indicating reduced inflammation. Hence, administration of MSCs inhibits the translocation, activity, and binding of NF-κB to DNA molecules, consequently lowering the inflammatory response in the liver following CLP.

### 3.6 MSCs, along with enhancement, restored the normal histology in hepatic tissues

**Figure 11:**
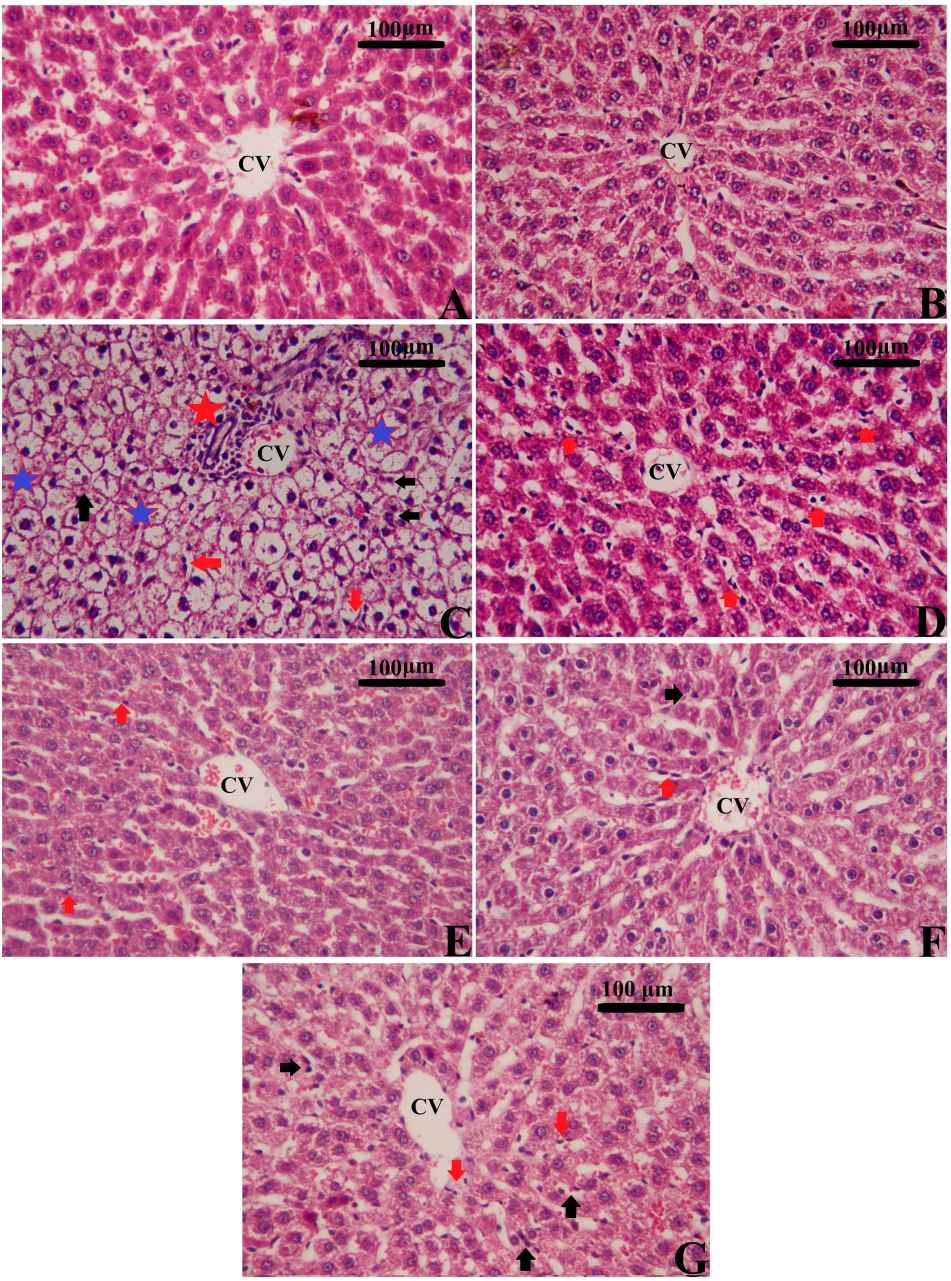
Effects of mesenchymal stem cells (MSCs), mesenchymal stem cells enhanced with Na_2_SeO_3_ (E1-MSCs), mesenchymal stem cells enhanced with SeNPs (E2-MSCs), and standard treatment antibiotics (Ab) on liver histopathology in CLP rats (H&E staining). Various magnifications were selected to illustrate typical tissue architecture and detailed histopathologic changes across broader areas. **(A)** Control; **(B)** Sham; **(C)** CLP; **(D)** CLP-MSCs; **(E)** CLP-E1-MSCs; **(F)** CLP-E2-MSCs; **(G)** CLP-Ab. CVs: central vein; black arrows: apoptotic nucleus; red arrows: inflammatory cells; red stars: focal inflammation; and blue stars: degenerative and vacuolated hepatocytes. Scale bar = 100 µm. Semi-quantitative analysis of histopathological changes is available in **Supplementary Data**, **Supplementary Figure S1**.

Rats with CPL-induced sepsis showed remarkable pathologic changes, including hepatocyte necrosis, architectural destruction, inflammatory cell infiltration, liver steatosis, and hepatic fibroplasia in the portal system when compared to the typical liver architecture of control rats using H&E-stained sections (**Figure 11**, **Supplementary Data**, **Supplementary Figure S1**). On the other hand, different treatments using MSCs alone or with Na_2_SeO_3_/SeNPs helped improve these changes in the liver’s histopathology, and the typical structure of the tissue was slowly restored. Both parts of the combination therapy restored liver histology and improved liver function by lowering serum transaminase levels. Our findings further highlight the significance of combined therapy as a novel therapeutic approach in regenerative medicine for treating sepsis-induced liver damage.

### 3.7 SeNPs preconditioning with MSCs lessened the hepatic apoptosis in septic rats

**Figure 12:**
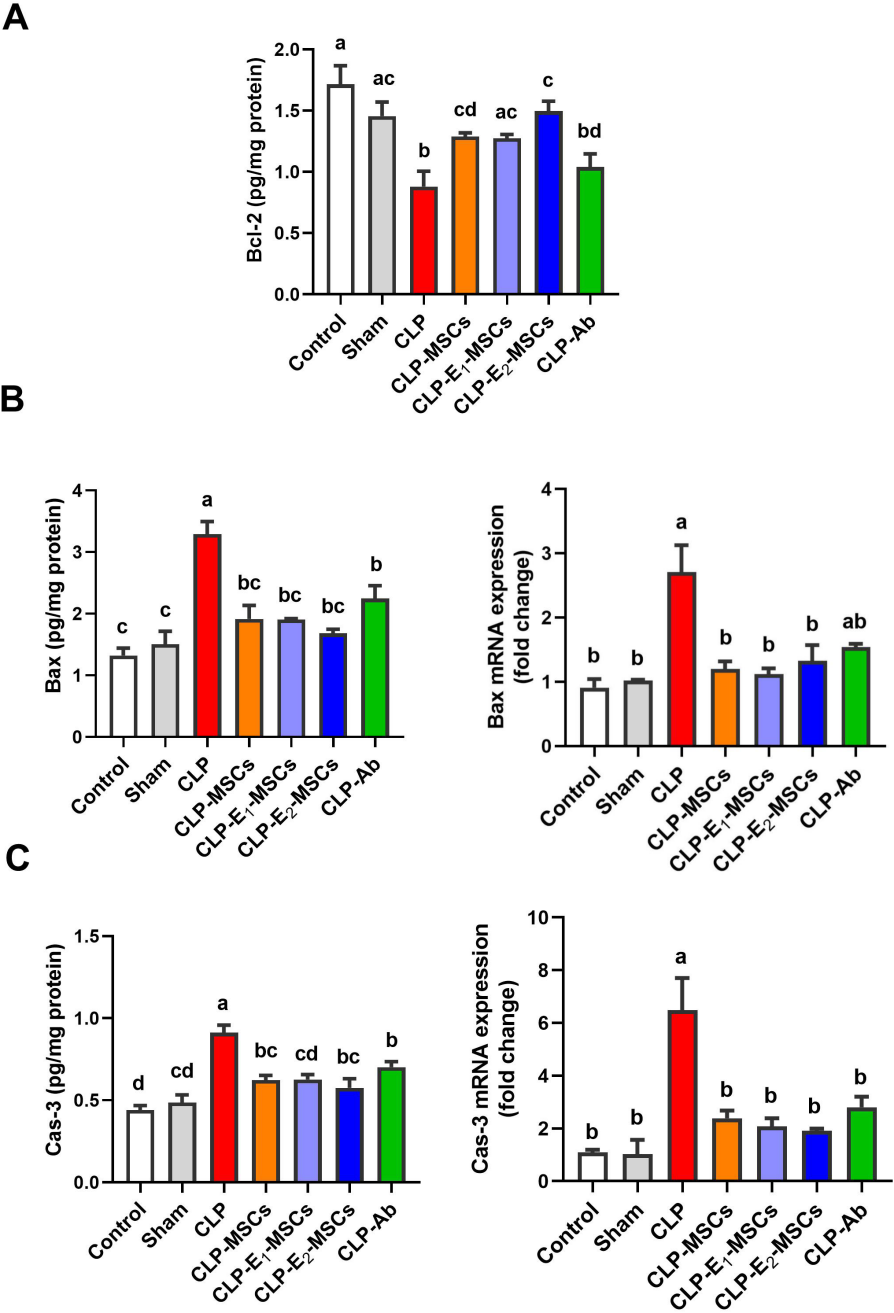
Effects of mesenchymal stem cells (MSCs), mesenchymal stem cells enhanced with Na_2_SeO_3_ (E_1_-MSCs), mesenchymal stem cells enhanced with SeNPs (E_2_-MSCs), or standard treatment antibiotics (Ab) on the apoptotic markers in damaged liver tissue of septic rats. **(A)** B-cell lymphoma-2 (Bcl-2), **(B)** bcl-2-associated X protein (Bax), and **(C)** caspase-3 (Cas-3). The mean ± SD data displays the biochemical results (n = 5), whereas gene expression findings are expressed as the mean ± SD of triplicate experiments adjusted to GAPDH. Different letters show statistically significant differences between groups by one-way ANOVA, with Tukey’s test at P < 0.05. Groups with the same letter have no significant differences.

To investigate hepatic apoptotic events in a rat model of CLP-induced sepsis and the possible anti-apoptotic impact of MSCs and their enhanced forms (E_1_-MSCs and E_2_-MSCs), hepatic tissue was analyzed for Bcl-2, Bax, and caspase-3 activity. Rats exposed to the CLP process exhibited significantly higher expression of genes of apoptogenic proteins [caspase-3 (P < 0.0001, F (6, 28) = 75.49) and Bax (P < 0.0001, F (6, 28) = 75.36)] than the control animal, while the level of the anti-apoptotic protein Bcl-2 was considerably lower (P < 0.0001, F = 38.63). In contrast to the untreated CLP levels, treatment with MSCs or their enhanced variants (E_1_-MSCs and E_2_-MSCs) significantly (P < 0.05) inhibited the apoptotic cascade. It reversed the changes in apoptotic proteins caused by CLP exposure, suggesting the protective role of MSCs alone or with enhancement against hepatocellular loss. Among the treatments, E_2_-MSCs demonstrated superior efficacy in restoring apoptotic markers, suggesting enhanced hepatoprotective properties. Based on these findings, E_2_-MSCs provide the most effective protection against CLP-induced hepatocellular apoptosis (**Figure 12**).

## 4 Discussion

Sepsis is still a serious issue in intensive care today, even with the newest medical advancements and medications. It could be life-threatening, leading to multiple organ failure. The inflammatory response to infection results in symptomatic changes in biology, physiology, and biochemistry, according to Vincent et al. (31). The cecal ligation and puncture model is considered the gold standard for researching sepsis in animals (32). The liver is vital for detoxifying and eliminating endotoxins during sepsis, as it produces inflammatory mediators and is an impacted organ (33). According to Yan et al. (34), reducing liver damage may reduce morbidity and death rates in sepsis patients. MSCs have been explored as a possible treatment for septicemia, considering their potential for regeneration, immunosuppressive features, immunomodulatory abilities, and anti-inflammatory qualities (35, 36). Under culturing conditions, oxidative stress can result in various molecular and cellular changes, including increased apoptosis, cell inactivation, phenotypic changes, diminished stemness and differentiation potential, and accelerated aging (9, 37). Researchers are investigating various approaches to combat oxidative stress and improve MSCs’ capacity to treat inflammatory disorders.

One such approach is adding antioxidants to the MSC culture medium (38). Selenium has protective properties for MSCs and can scavenge ROS (39, 40). It serves as an enzyme cofactor for GPX and TrxR as well as an antioxidant. Moreover, it is involved in redox regulation, and the selenium nanoform can enhance the proliferation of stem cells (39). In the current research, we investigated the possible function of MSCs alone or enhanced with sodium selenite (Na_2_SeO_3_, which is E_1_-MSCs) or selenium nanoparticles (SeNPs, which are E_2_-MSCs) in a CLP rat model. The synthesized SeNPs (**Figures 2A, B**) exhibited a uniform size distribution (PDI = 0.068) and high stability (zeta potential = 19.5 ± 0.11 mV) and were subsequently utilized to enhance MSCs. MSCs’ therapeutic potential was ensured by flow cytometry, which verified their purity by showing that 82.5% of cells expressed CD44 but lacked CD45/CD19 (**Figures 3B–D**). Our research revealed that MSCs (both alone and enhanced) have promising effects on sepsis. Reducing pro-inflammatory mediators and cytokines, preventing liver damage, reducing oxidative stress, strengthening the antioxidant defense system, significant immunomodulation, and preserving vital tissues are considered potential roles of MSCs—an overall reduction in the morbidity and death caused by sepsis.

Regarding the hepatoprotective effects of MSCs alone or enhanced with Na_2_SeO_3_ (E1-MSCs) or SeNPs (E2-MSCs) in treating changes observed in septic rats, our findings indicate significant alterations in liver tissues due to CLP, evident at both histological and biochemical levels. Our histological examination verified the hepatic damage, as CLP led to the emergence of steatosis, hepatocellular necrosis, significant inflammatory cell infiltration, central vein dilatation and congestion, and Kupffer cell hyperplasia (**Figure 10**). These results align with earlier research (7, 19, 41). Biochemically, the serum levels of ALT, AST, and ALP were significantly elevated, confirming the extensive liver damage caused by CLP. Luo et al. (19) and Jeschke {Jeschke, 2009 #25794} reported that these hepatic enzymes are cytoplasmic in origin, but when a liver injury occurs, they leak into the bloodstream, reflecting a loss of the liver’s functional integrity. Other researchers (42, 43) also reported comparable results. Fortunately, in the current study, treating CLP-induced sepsis in liver tissue with MSCs alone or combined with sodium selenite (E_1-_MSCs) or selenium nanoparticles (E_2_-MSCs) has significantly improved the liver’s overall structural and functional alterations, which is consistent with the earlier study by Selim et al. (44). Following treatment, liver tissue revealed nearly standard architecture, reduced hepatocyte necrosis and fatty alterations, and lessened central hepatic vein enlargement and congestion. Hepatic enzyme levels (**Figure 4**; ALT, AST, and ALP) significantly decreased after treatment, indicating potential hepatoprotective benefits of MSCs on their own or with enhancement against the CLP septic model (19). Similarly, Zhao et al. {Zhao, 2012 #25798} previously showed that bone marrow-derived stem cells can lower serum transaminases in chemically induced acute liver injury. According to our research, MSCs alone or in combination with selenium-enhancing substances may be a viable treatment option for liver damage brought on by sepsis. In line with their established antioxidant and immunomodulatory functions, these histological and biochemical advancements confirm that SeNPs-MSCs simultaneously target inflammation, oxidative stress, and tissue damage. More studies are required to clarify the precise mechanisms underlying these hepatoprotective effects and to improve treatment regimens.

Sepsis-induced hepatic impairment is caused mainly by elevated oxidative stress (45). In line with earlier studies (46, 47). We noticed elevated lipid peroxidation, GSH depletion, and suppression of antioxidant enzyme activity in the septic rat group, confirming a state of severe oxidative stress. These alterations are due to the adverse effects triggered by ROS (reactive oxygen species) and RNS (reactive nitrogen species) on the mitochondria of hepatocytes, which can potentially result in apoptosis, necrosis, and liver failure (48, 49).

Malondialdehyde is a byproduct of lipid peroxidation and a recognized indicator of oxidative stress. It is also a typical indicator of elevated oxidative stress (50, 51). Simultaneously, GSH depletion is crucial for maintaining the stability of lipids and proteins and keeping cellular redox balance (52, 53). Additionally, superoxide dismutase, catalase, and glutathione peroxidase impairment have been shown to support the overloaded antioxidant system in septic livers (33, 54). These outcomes reveal the potential therapeutic benefits of antioxidant therapies for the management of impairment of the liver caused by sepsis.

Our research demonstrated that all treatment groups had a restricted level of lipid peroxidation (**Figure 5A**), significantly higher GSH content, and antioxidant enzyme activities (**Figure 6**). This is consistent with MSCs’ well-established antioxidant properties, which, as reported by Stavely and Nurgali (55), include the ability to directly scavenge reactive oxygen species (ROS) and donate functioning mitochondria or indirectly stimulate the antioxidant systems in other cells and modulate the bioenergetics. The MSC treatment in our study—whether used alone or with Na_2_SeO_3_ or SeNPs—worked better than regular antibiotics at lowering oxidative damage. The improved effectiveness might come from the combined protective benefits of MSC-derived antioxidants and selenium’s ability to boost MSC function, as shown by Rahimi et al. (56). Further, these effects positively correlate with the MSC-induced recovery in histopathology and liver functioning. Restoring redox balance likely helps explain the overall benefits of MSCs in treating sepsis, as oxidative stress can worsen organ failure and inflammation. Our findings show that MSCs have two important roles: they help reduce ROS and improve the body’s antioxidant systems, which explains how they protect against sepsis.

Moreover, sepsis induces parenchymal cells and vascular dysfunction, which leads to neutrophils infiltrating the site of damage or infection (42). Myeloperoxidase also indicates increased neutrophils and inflammatory cell infiltration (57). The production of MPO and other molecules by activated neutrophils leads to the overproduction of ROS (58). The current study revealed a significant rise in MPO activity, which implies a prominent infiltration of neutrophils and ongoing inflammatory processes in the hepatic tissue of the CLP rats. At the same time, MPO activity significantly decreased due to MSC administration (59, 60). Interestingly, our study introduced a novel approach of combining MSCs with SeNPs to improve the outcome. The combined therapy group (E_2_-MSCs) has shown a significant decrease in MPO activity, suggesting a substantial reduction in neutrophil infiltration and liver damage. Further evidence to support the result was obtained from histopathological investigation, which showed a decrease in neutrophil infiltration in the hepatic tissues in the combined therapy group. The correlation between MPO activity and histological changes highlights the potential of combined therapy for MSCs and SeNPs to attenuate sepsis-induced liver damage, underscoring the relevance of our findings.

Sepsis induces a complicated, inflammatory response involving infiltrating immune cells and liver resident cells. According to Yan et al. (34), these cells produce a variety of inflammatory mediators that contribute to organ damage caused by sepsis, such as TNF-α, interleukin-1β, interleukin-8, nitric oxide, and ROS. TNF-α is believed to be an essential mediator in sepsis, primarily generated by Kupffer cells (61). Septic patients and animal models generally show elevated levels (62). TNF-α stimulates specific transmembrane receptors, resulting in immune cell activation and the subsequent release of chemicals causing inflammation. This cycle may result in oxidative stress-induced DNA damage (33). Like TNF-α, IL-1β is produced by activated macrophages and is essential for numerous cellular processes, including differentiation, apoptosis, and proliferation (63). IL-8, another chemokine also generated by macrophages and other cells, acts as a neutrophil chemotactic agent, attracting neutrophils to the site of inflammation. Elevated IL-8 levels have been detected in patients with sepsis (64). Our findings revealed that pro-inflammatory cytokines (TNF-α, IL-1β, IL-8) significantly increased in septic rats, indicating systemic inflammation. Immunohistochemistry revealed TNF-α upregulation in hepatic cells, featuring strong staining in cytoplasm and nuclei, indicating serious tissue injury. Nevertheless, the injection of MSCs, either alone or in combination with selenium (especially as E2-MSC nanoparticles), showed a notable decrease in cytokine levels and limited TNF-α to the cytoplasm (IHC), demonstrating the anti-inflammatory and tissue-regeneration abilities of MSCs and combined therapy (65, 66). Moreover, the study of Abdel-Salam et al. (67) showed that AT-MSCs decrease TNF-α immunoreactivity in the liver tissues compared to the LPS control group. The coordinated inhibition of cytokines (IL-1β/IL-8) and pathogenic TNF-α translocation demonstrates MSCs’ ability to combat inflammation at both systemic and cellular levels. Given the strong relationship between inflammation and oxidative stress in sepsis, the observed decrease in oxidative damage could account for the concurrent decline in pro-inflammatory markers, as demonstrated by our findings (**Figures 7A–C**). This evidence indicates that MSCs reduce oxidative injury and may play a role in overall tissue protection by interrupting the cycle of oxidative stress and inflammation.

The study also observed a marked reduction in hepatic levels of NO and gene expression of inducible nitric oxide synthase in the CLP groups receiving MSC therapy. Pro-inflammatory cytokines like TNF-α and IL-1β cause sustained large quantities of NO generation via stimulating iNOS, according to Kamanaka et al. (68) and Celep and Gedikli (69). This excess NO may disrupt endothelial, neuronal, and epithelial cells, resulting in liver damage, according to a study by Hua et al. (70) who showed that amnion-derived MSCs (A-MSCs) restricted iNOS activity, NO production, and inflammatory cytokine synthesis while triggering an anti-inflammatory response in liver macrophages. Selenium supplements efficiently inhibit cytokine-induced NF-κB and iNOS upregulation, reducing NO levels (71, 72). Selenium can control NO generation through GPx’s inhibitory effect on iNOS expression (73). Similarly, our results demonstrate that the co-administered therapy attained recovery from hepatic inflammation and maintained redox equilibrium.

In septic rats, we observed significant NF-κB activation, characterized by increased levels measured by ELISA (**Figure 7F**) and intense nuclear and cytoplasmic staining via immunohistochemistry (**Figures 8**, **9**), confirming its involvement in mediating inflammation. The NF-κB nuclear translocation is crucial for upregulating pro-inflammatory genes (e.g., TNF-α, IL-1β) and ROS-producing enzymes, worsening tissue damage. This supports NF-κB’s known role in regulating cytokine gene expression (74). MSCs have anti-inflammatory effects on acute liver injury by inhibiting the active NF-κB pathway, an essential mediator during serious infections (75). Our results showed that MSC alone or with enhancement treatment decreased NF-κB protein levels and inhibited its nuclear translocation (IHC), localizing it to the cytoplasm. Our findings align with those of previous authors who suggested that MSC-driven suppression of the NF-κB pathway is a crucial therapeutic approach for liver damage resulting from sepsis (76). Additionally, selenite-cultured cells have demonstrated that selenium reduces NF-κB activation and modifies the expression of inflammatory cytokine genes (77). This suppression was associated with reduced oxidative stress (lower lipid peroxidation, restored GSH) and diminished cytokine release, indicating that MSCs interrupt the NF-κB-mediated connection between inflammation and ROS generation. In summary, administration of MSCs, either by themselves or in cooperation with SeNPs (E2-MSCs), assists in keeping pro- and anti-inflammatory cytokines in balance in septic rats (78).

According to our research, MSCs and Na_2_SeO_3_, or SeNPs, work together synergistically to modulate the immune system and reduce inflammation in the hepatic tissues of septic rats. Even though the inflammatory response is essential for struggling with infection, an overreaction to it in late sepsis can inhibit the immune system and cause multiple organ failure or even death (79). Regarding potential mechanisms of inflammation, the newest research has shown the significance of cyclooxygenase-2 in producing excessive amounts of prostaglandin E2 during the inflammatory process (80). The pathway that typically controls the release of COX-2 in inflammation involves the mitogen-activated protein kinases (81). The MAPK subgroup JNK2 isoform (MAPK9) mainly engages in inflammation and liver metabolism. Moreover, its upregulation induces neutrophils and macrophages to infiltrate the liver and produce inflammatory cytokines (82). Our findings support previous reports (83); notable decreases were observed in levels of COX-2 and PGE2, as well as a noteworthy downregulation in Mapk9 gene expression in hepatic tissues treated with MSCs in either mono- or combined therapy (E_1_-MSCs/E_2_-MSCs). The positive outcomes of MSCs were mainly related to paracrine behaviors, releasing signaling factors for tissue regeneration (84, 85). Prostaglandin E2, one of these paracrine impacts, is an immunosuppressive cytokine that regulates inflammation and immunological responses (60).

Additionally, MSCs inhibit dendritic cells’ capacity to activate T cells, interfere with their differentiation, maturation, and function, and generate cytokines that are more anti-inflammatory and less pro-inflammatory (86, 87). Consequently, MSCs inhibit the MAPK pathway to reduce the production of COX-2 and other inflammatory mediators. Preserving the immune system’s stability and piercing tissues and organs to perform further functions (88). Furthermore, insufficient selenium may impair selenoprotein expression and GPx activity, indirectly affecting COX expression through the MAPK pathway and COX-2 (89). Conversely, selenium supplementation boosts anti-inflammatory mechanisms by reducing these intermediaries, which can mitigate these inflammatory alterations (90, 91). It is crucial to observe that preconditioning SeNPs for MSCs significantly reduced the production of pro-inflammatory cytokines in the hepatic tissue compared to MSCs alone, suggesting that SeNPs have enhanced the anti-inflammatory effects of MSCs.

Hepatocellular apoptosis spurred by sepsis induces organ malfunction and destruction (92). According to Fu et al. (93) and Klingensmith et al. (94), Bcl-2 and Bax are essential mediators of the apoptotic process. Also, it is characterized by cleaved caspase-3 (95). Consequently, apoptosis regulation suggests a therapeutic strategy for preventing liver damage (33). Our findings showed that sepsis markedly increased the liver’s pro-apoptotic pathways, as evidenced by increased protein levels of the proteins Caspase-3 and Bax and RT-PCR production of their related mRNA (**Figures 11B, C**). On the other hand, a shift toward apoptosis was shown by a substantial decrease in Bcl-2 protein levels (**Figure 11A**). MSC treatment effectively addressed this imbalance, normalizing the expression of Caspase-3 and Bax at both the protein and mRNA levels, while also restoring Bcl-2 protein levels. This is consistent with earlier research indicating that MSCs inhibit apoptosis by modulating pro- and anti-apoptotic factors (96, 97), and the MSCs can treat various illnesses by boosting tissue cell proliferation and blocking atypical apoptosis (98, 99). Similarly, selenium can alleviate cardiovascular diseases by modulating apoptosis, elevating the expression of the anti-apoptotic Bcl-2, and correcting the elevated levels of the pro-apoptotic Bax and Caspase-3 (100, 101). Furthermore, selenium shields cells against immune suppression, cytotoxicity, and intrinsic apoptosis by scavenging ROS (102, 103). These results indicate that MSCs, especially when combined with selenium, regulate multiple important apoptotic pathways to decrease sepsis-induced apoptosis and contribute to the simultaneous reduction of oxidative damage and inflammation, suggesting an interconnected protective mechanism.

## 5 Conclusions

According to the study’s findings, sepsis-induced acute liver damage is probably caused by a variety of processes. This study showed that the combination of Na_2_SeO_3_ or SeNPs-MSCs is more effective than either treatment alone in improving sepsis-induced acute liver injury, hepatic function, and outcomes in a rodent model. This was primarily due to the reduction of fibrosis and apoptosis, the suppression of ROS generation and oxidative stress, the inhibition of inflammation and inappropriate immune response, the upregulation of antioxidant expressions and potential liver-organ regeneration, and the preservation of hepatic architecture. These findings have crucially clarified the therapeutic potential of Na_2_SeO_3_/SeNPs and MSC combination therapy in reducing liver damage brought on by CLP.

## Data Availability

The original contributions presented in the study are included in the article/**Supplementary Material**. Further inquiries can be directed to the corresponding author.
